# Endometrial Thickness as a Prognostic Marker in Premenopausal Abnormal Uterine Bleeding: Diagnostic Accuracy and Kaplan–Meier Survival Insights

**DOI:** 10.12688/f1000research.170554.2

**Published:** 2025-12-31

**Authors:** Narjes karmous, Hanene Jemli, Abdelwahab Masmoudi, Badreddine Bouguerra, Aymen Mabrouk, Anis Ben Dhaou, Abdennour Karmous

**Affiliations:** 1 Gynaecology and Obstetrics department B, Charles Nicolle Hospital, Tunis, Tunisia; 2 Faculty of medicine of Tunis, University Tunis el Manar, Tunis, Tunisia; 3 General surgery department B, Charles Nicolle Hospital, Tunis, Tunisia; 4 Psychiatric department, Razi Hospital, Tunis, Tunisia

**Keywords:** Premenopausal women; abnormal uterine bleeding; transvaginal ultrasound; endometrial thickness, endometrial cancer; diagnostic accuracy

## Abstract

**Background:**

Abnormal uterine bleeding (AUB) in premenopausal women is frequent, yet distinguishing benign from malignant causes remains clinically challenging. Endometrial thickness (ET) measured by transvaginal ultrasound (TVUS) is well established in postmenopausal bleeding but less validated in premenopausal women due to cyclical variations. This study aimed to assess the diagnostic accuracy and prognostic value of ET, supplemented by Kaplan–Meier survival analysis, in predicting endometrial malignancy among premenopausal women with AUB.

**Methods:**

We conducted a retrospective analytical study including premenopausal women presenting with AUB at Charles Nicolle Hospital, Tunis, Tunisia (2016–2024). All underwent TVUS followed by endometrial sampling (hysteroscopy or curettage) and definitive histological confirmation on hysterectomy specimens. Sonographic features were described using International Endometrial Tumor Analysis (IETA) criteria. Diagnostic performance of ET was evaluated using receiver operating characteristic (ROC) curve analysis. Kaplan-Meir survival analysis was applied to time-to-consultation and time-to-diagnosis, not survival outcomes.

**Results:**

ROC analysis identified ET as the strongest predictor of endometrial malignancy (AUC = 0.842, p < 0.001). An optimal cutoff of >9 mm achieved 69.2% sensitivity, 87.1% specificity, and a negative predictive value of 97.7%, effectively ruling out malignancy in patients with ET ≤9 mm. Although the positive predictive value was modest (26.5%), ET reliably stratified risk for further invasive evaluation. Kaplan–Meier analysis demonstrated that ET >9 mm was associated with earlier consultation (mean 27.7 vs. 70.3 months, p < 0.0001) and shorter time-to-diagnosis (median 12 months vs. not reached, p < 0.0001), reflecting a more aggressive clinical course.

**Conclusions:**

ET >9 mm is a robust, non-invasive potential prognostic marker in premenopausal AUB, identifying high-risk women requiring urgent evaluation while safely excluding malignancy in low-risk patients.

## 1. Introduction

Abnormal uterine bleeding (AUB) is a prevalent gynecological concern in premenopausal women. ^1^ While the majority of cases stem from benign conditions, the potential for endometrial hyperplasia (EH) or malignancy necessitates a careful diagnostic approach. ^2, 3^

In postmenopausal women, transvaginal ultrasound (TVUS) measurement of endometrial thickness (ET) is a well-validated tool, with an ET <4 mm effectively ruling out cancer in most cases. ^4– 6^ However, this single metric is of limited value in premenopausal women due to normal cyclical hormonal variations that cause ET to fluctuate significantly. This has created a diagnostic gap, leading to reliance on clinical risk factors (e.g., obesity, anovulation) for deciding on invasive endometrial sampling. ^7, 8^ The limitations of this approach underscore the critical need for more objective, imaging-based criteria.

This study aimed to evaluate the diagnostic and prognostic value of ET in premenopausal women with abnormal uterine bleeding. We assessed the accuracy of ET for predicting malignancy, determined the optimal cutoff, and used Kaplan–Meier analysis to examine its impact on the timing of consultation and definitive diagnosis.

## 2. Methods

### 2.1 Study design and setting

A retrospective, longitudinal and analytical study was carried out over an 8- year and 11-month, from January 2016 to November 2024, at Gynaecology and Obstetrics Department B, Charles Nicolle Hospital, Tunis, Tunisia.

### 2.2 Study population

Premenopausal women presenting with AUB who underwent TVUS followed by endometrial biopsy (via hysteroscopy or curettage) and subsequent definitive surgical treatment (hysterectomy) were identified.

AUB refers to bleeding from the uterine corpus that is abnormal in regularity, volume, frequency or duration. It encompasses heavy menstrual bleeding, irregular menstrual bleeding and intermenstrual bleeding. ^9^

The ultrasound features of different endometrial and other intracavitary pathologies were assessed using the International Endometrial Tumor Analysis (IETA) terminology. ^10^

TVUS were performed by three experienced operators, all holding an inter-university diploma in gynecologic ultrasound and each having over 10 years of clinical experience in pelvic ultrasound practice.

The study population consisted of patients who fulfilled the following criteria:

▪ **Inclusion Criteria**:
  − Presence of AUB.
  − Available TVUS report with detailed description of endometrial features.
  − Final histological diagnosis on hysterectomy specimen.
▪ **Exclusion Criteria**:
  − Pregnant women.
  − Incomplete TVUS data or poor image quality.
  − No subsequent hysterectomy performed.

**Figure 1.**
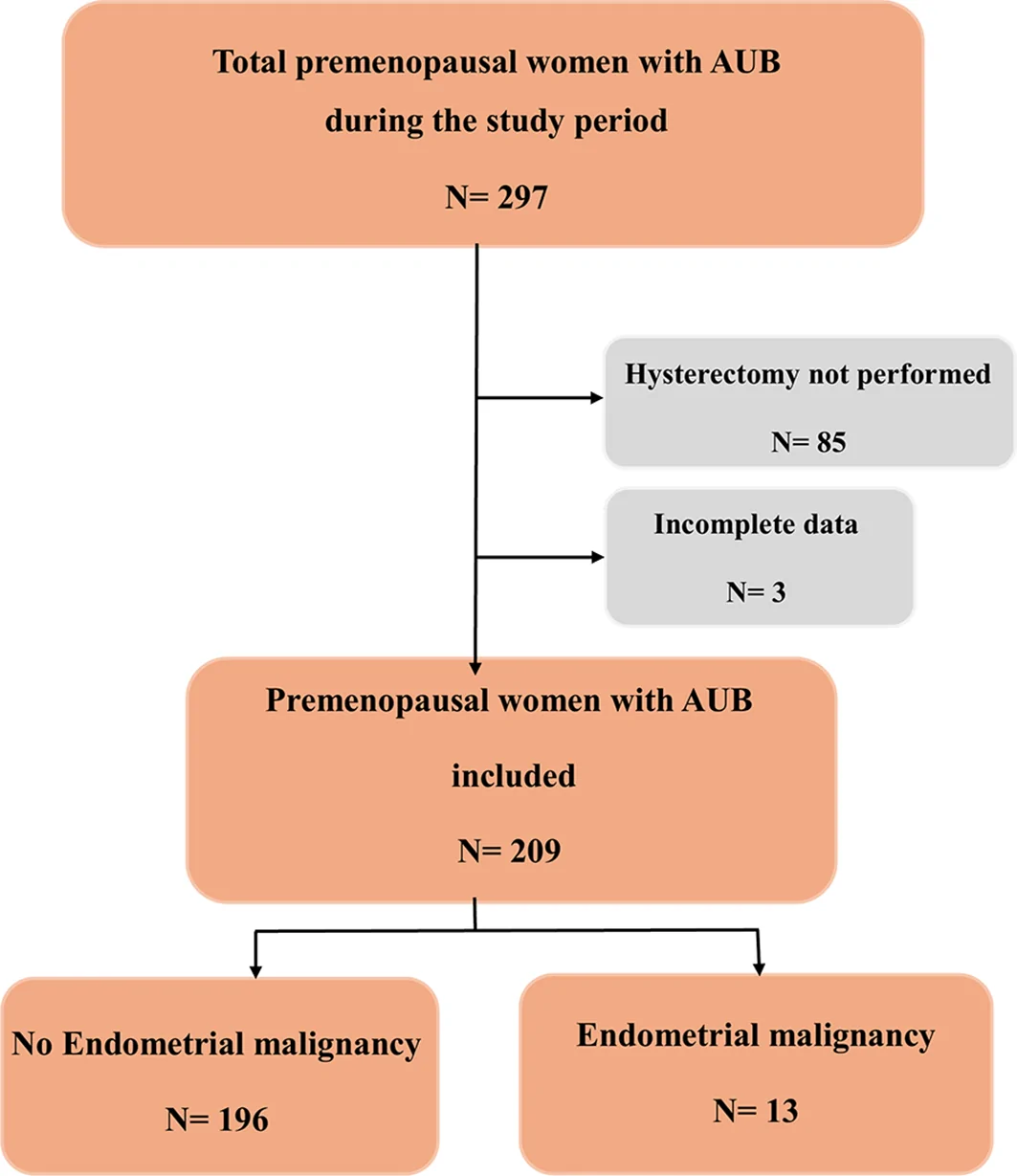
Flowchart of the study population.

A study flowchart detailing case selection and exclusions has been developed ( Figure 1 **)**.

### 2.3 Variables

Data were collected from patient records onto a pre-established form.

**2.3.1 Patients’ characteristics**

Age, family and personal history, AUB (type, abundance, number of haemorrhage episodes, associated symptoms …), delay before consultation, time to diagnosis …

**2.3.2 TVUS features**

The primary data extracted from the TVUS reports included ^10^:

▪ **Endometrial thickness:** ET was measured in the sagittal plane as the double-layer distance between the two endometrial–myometrial interfaces, at the point of greatest thickness perpendicular to the midline, using appropriately magnified images. The result was reported in millimetres.
▪ **Endometrial-myometrial junction:** Classified as regular, irregular, interrupted or not defined.
▪ **Intracavitary images:** Note of any focal lesions, such as polyps or masses, intracavitary fluid.
▪ **Uterine Size:** Measurement of uterine dimensions.
▪ **Endometrial vascularity:** Color Doppler assessment of the endometrium: a color score of 1 was given to indicate no color (no flow); a score of 2 indicates minimal color (minimal flow) and a score of 3 indicates moderate color (moderate flow).
▪ **Associated Features:** Presence of fibroids, adenomyosis, or ovarian cysts.

**2.3.3 Uterine cavity exploration**

Hysteroscopy or curettage, date of endometrial sampling, abundance of the sample, anatomopathological diagnosis from the biopsy.

**2.3.4 Definitive anatomopathological diagnosis**

The definitive histological diagnosis on the hysterectomy specimen was considered the reference standard.

### 2.4 Statistical analysis

All analyses were performed using IBM SPSS Statistics (Version 26.0; IBM Corp, Armonk, NY, USA) for data management, descriptive statistics, correlation analysis, and non-parametric tests.

For comparative analysis, variables significantly associated with endometrial malignancy were assessed using the chi-square test or Fisher’s exact test for categorical variables and Student’s t test or Mann-Whitney U test for continuous variables.

Multivariate logistic regression models were constructed to identify independent predictors of endometrial malignancy cases. Variables with a p value ≤ 0.20 in the univariate analysis were included in the model. Adjusted odds ratios (ORs) and 95% confidence intervals (CIs) were reported. A p value ≤ 0.05 was considered statistically significant. ^11, 12^

These variables comprised: oral contraceptive use, previous uterine endoscopic procedure, number of hemorrhage episodes, vascularization score, bleeding abundance, and endometrial thickness >9 mm (See Table 1).

**Table 1.** Variables included in the multivariate logistic regression model.

Type of variable	Variables included in multivariate model
Clinical	Oral contraceptive use, Number of hemorrhage episodes, Bleeding abundance
Procedural	Previous uterine endoscopic procedure
Sonographic	Vascularization score, Endometrial thickness >9 mm

The diagnostic performance of each sonographic feature for predicting endometrial malignancy was assessed using sensitivity, specificity, positive predictive value (PPV), and negative predictive value (NPV). ^13^

A Kaplan-Meier survival analysis was conducted to assess the prognostic value of ET for predicting the timing of a malignant diagnosis. ^14^

### 2.5 Ethical considerations

The study protocol received approval from the institutional ethics committee of Charles Nicolle Hospital, Tunis, Tunisia on 6 Mars 2025 by before conducting the study (approval number FWA 00032748- IORG0011243).

Given the retrospective design and the use of anonymized data, the committee granted a waiver of informed consent.

## 3. Results

The study included 209 patients. Final histopathological examination revealed endometrial malignancy in 13 premenopausal women (6.2%), while 196 women (93.8%) had benign endometrium.

### 3.1 Comparative study

The median age was 46 years in benign cases and 48 years in malignant cases (p = 0.217).

The median BMI was 25 for benign and 26 for malignant cases (p = 0.576).

Most patients had no family cancer history (94% benign vs. 6% malignant, p = 0.123).

Diabetes (93% benign vs. 7% malignant, p = 0.338) and hypertension (94% benign vs. 6% malignant, p = 0.954) were uncommon.

Median age at menarche was 12 years in both groups (p = 0.603).

Gravidity and parity differed, with median gravidity of 3 versus 2 (p = 0.018) and median parity of 3 versus 2 (p = 0.060).

Breast neoplasia, tamoxifen use, and pelvic irradiation were not associated with malignancy.

Bleeding characteristics—including abundance and type—showed limited association, except for the number of hemorrhage episodes (p = 0.027), where patients with 0–1 episode had higher malignancy rates (25–33%) than those with ≥2 episodes.

The median delay before consultation was significantly shorter in malignant cases (6 months) compared to benign cases (12 months, p = 0.011).

Regarding ultrasound features ( Table 2), ET was markedly higher in malignant cases (median 12 mm) than in benign cases (median 5 mm, p < 0.001). Vascularization (p < 0.001), with Score 2 showed 71% malignancy, whereas normal vascularization or Score 1 had much lower rates. The uterine size, the endometrial–myometrial interface and intracavitary images did not show significant differences. Ovarian cysts did not show a significant association with malignancy (p = 0.808).

**Table 2.** Ultrasound features among the study population.

Variable	Category	Benign n (%)	Malignant n (%)	Total n (%)	p-value
**Endometrial thickness (mm)**	-	5	10	-	**0.000**
**Uterine size**	**Normal**	45 (94)	3 (6)	48 (100)	0.992
	**Increased**	151 (94)	10 (6)	161 (100)	
**Vascularization**	**No**	97 (98)	2 (2)	99 (100)	**0.000**
	**Score 1**	97 (94)	6 (6)	103 (100)	
	**Score 2**	2 (29)	5 (71)	7 (100)	
**Endometrial–myometrial interface**	**Unseen**	84 (93)	6 (7)	90 (100)	0.828
	**Seen**	111 (94)	7 (6)	118 (100)	
**Intracavitary image**	**No**	149 (93)	11 (7)	160 (100)	0.871
	**Polyp**	15 (94)	1 (6)	16 (100)	
	**Fibroma**	31 (97)	1 (3)	32 (100)	
	**Other**	1 (100)	0 (0)	1 (100)	
	**Profuse**	114 (90)	13 (10)	127 (100)	

Presence of fibroids was associated with a lower risk of malignancy (3.8% malignant in patients with fibroids vs. 14% in those without, p = 0.009).

Hemostatic curettage, and hysteroscopy did not show significant differences. Quantity of curettage specimen was significant (p = 0.003), with abundant specimens associated with 10% malignancy compared to 0% for scanty specimens.

### 3.2 Receiver operating characteristic (ROC) and logistic regression analyses ( Figure 2, Table 3)

**Figure 2.**
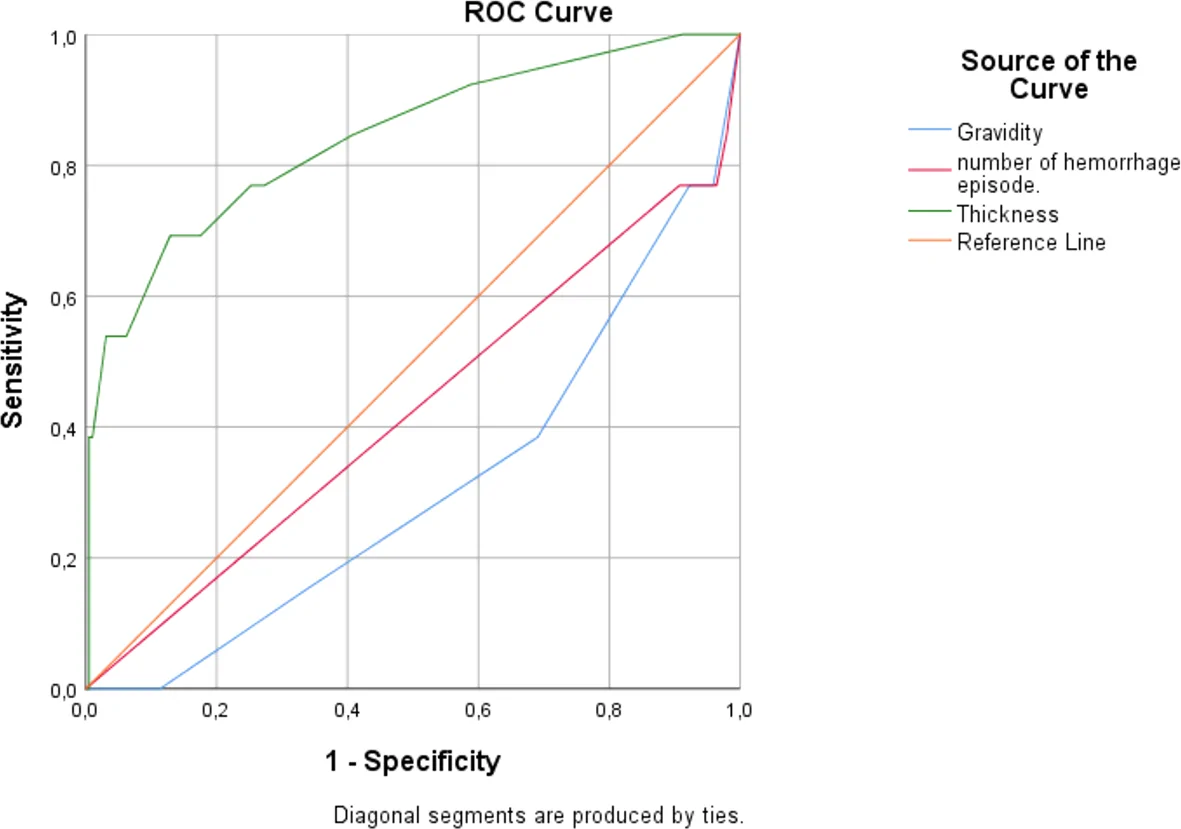
Predictive performance of endometrial thickness versus clinical features (gravidity, number of hemorrhage episodes) for endometrial malignancy based on ROC analysis.

**Table 3.** Predictive performance of endometrial thickness comparatively with clinical features (gravidity, number of hemorrhage episodes) for endometrial malignancy was evaluated using ROC analysis.

Variable	AUC	p-value	95% CI	Criterion	Sensitivity (%)	Specificity (%)	PPV ^*^ (%)	NPV ^**^ (%)
**Gravidity**	0.690	0.022	0.542–0.838	≤1	23.08	96.43	30	95
**Number of hemorrhage episodes**	0.576	0.360	0.398–0.754	≤2	61.54	68.88	11.6	96.4
**Endometrial thickness**	0.842	<0.001	0.715–0.968	>9	69.23	87.11	26.5	97.7

^*^ PPV: Predictive positive value.

^**^ NPV: Negative predictive value.

ET measured via TVUS was unequivocally identified as the single most robust independent predictor of endometrial malignancy within our studied cohort. Its superior performance, quantified by an excellent Area Under the Curve (AUC) of 0.842 (p < 0.001), underscores its unparalleled utility in the initial triage of patients presenting with AUB.

This diagnostic power significantly surpassed that of other clinical variables, such as gravidity (AUC = 0.690) and the number of hemorrhage episodes (AUC = 0.576, nonsignificant), establishing ET as the cornerstone of non-invasive risk assessment.

Variables showing an association with malignancy in univariate analysis (p < 0.10) were included in a multivariate logistic regression model. The final model identified several independent predictors of malignant endometrial pathology ( Table 4).

**Table 4.** Independent predictors of endometrial malignancy identified by logistic regression.

	p-value	OR	95% CI for OR	
			Lower	Upper
**Oral contraceptive use**	0.025	29.866	1.519	587.080
**Previous uterine endoscopic procedure**	0.054	6.180	0.970	39.392
**Number of hemorrhage episode ≤1**	0.001	0.202	0.079	0.520
**Vascularization**	0.006	98.343	3.727	2594.786
**Thickness >9**	<0.001	25.254	4.320	147.642
**Bleeding abundance**	0.002	0.295	0.134	0.648

The derivation of an optimal cut-off >9 mm provides a clear, evidence-based threshold for clinical decision-making. This criterion successfully balances sensitivity (69.2%) and specificity (87.1%), ensuring a high detection rate for malignancies while effectively minimizing false alarms.

The specificity of 87.1% is particularly noteworthy; it signifies that most patients with benign conditions can be correctly identified by this simple measure, preventing anxiety and unnecessary procedures.

However, the most profound clinical implication of our findings lies in the metric of the NPV, which reached 97.7%. This is arguably the most significant result of the analysis. It translates to a powerful clinical rule: an endometrial measurement of ≤9 mm effectively rules out malignancy with a certainty of over 97%. This provides immense reassurance to both the clinician and the patient, creating a safe pathway to conservatively manage a large portion of low-risk individuals. It can justify a strategy of watchful waiting or medical management for these patients, thereby reducing the number of invasive, costly, and potentially uncomfortable procedures like hysteroscopy or dilation and curettage.

Conversely, the PPV of 26.5% for a thickness >9 mm contextualizes its use. It confirms that while a thick endometrium is a strong risk marker mandating histological sampling, it is not diagnostic of cancer on its own. Most positive tests (73.5%) will be due to benign conditions like hyperplasia or polyps. Therefore, the role of ET is not to definitively diagnose cancer but to act as an exceptionally efficient screening gatekeeper—identifying all high-risk patients who require definitive biopsy while safeguarding low-risk patients from undue intervention.

### 3.3 Kaplan-Meier survival curve analysis

To assess the prognostic value of ET for predicting the timing of a malignant diagnosis, two Kaplan-Meier survival analyses were conducted:

- The first analysis evaluated the time from symptom onset to initial consultation: **time-to-consultation.**
- The second evaluated the time from initial consultation to definitive histological diagnosis: **time-to-diagnosis**.

The cohort was stratified by an ET cutoff of >9 mm:

- The group with an ET ≤9 mm (n = 175), 4 (2.3%) received a final histological diagnosis of malignancy.
- Conversely, in the group with an ET >9 mm (n = 34), 9 patients (26.5%) were diagnosed with malignancy, underscoring a substantially higher disease prevalence in this group.

**3.3.1 Time-to-consultation analysis**

**Figure 3.**
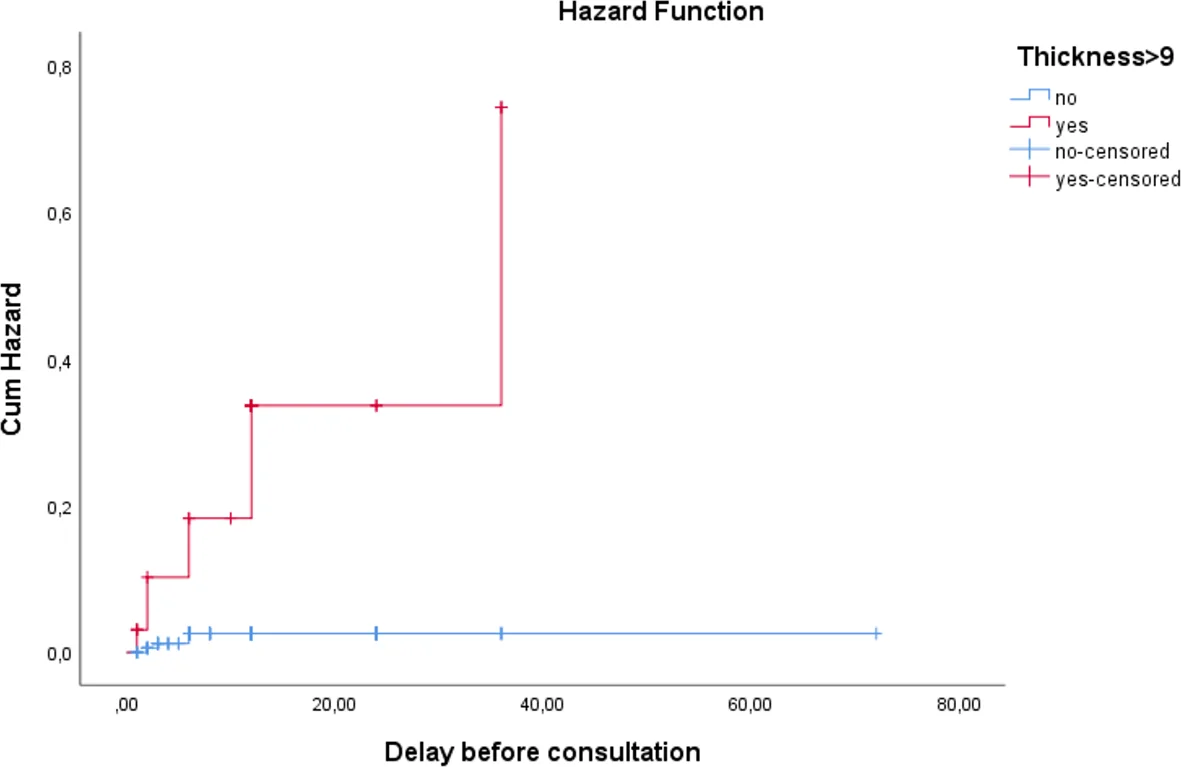
Kaplan–Meier survival analyses: time from symptom onset to initial consultation (time-to-consultation).

The interval from symptom onset to first consultation differed significantly between groups (Log-rank test χ ^2^ = 25.662, p < .0001): ( Figure 3)

- The estimated mean time-to-consultation was more than 2.5 times longer for patients with an ET ≤9 mm (70.3 months, 95% CI: 68.6 – 71.9) compared to those with an ET >9 mm (27.7 months, 95% CI: 22.5 – 32.9).
- The median time-to-consultation was 36 months for the high-ET group and was not reached for the low-ET group.

This indicates that symptoms associated with a thicker endometrium (e.g., heavier bleeding) prompt patients to seek medical attention much sooner.

**3.3.2 Time-to-diagnosis analysis**

**Figure 4.**
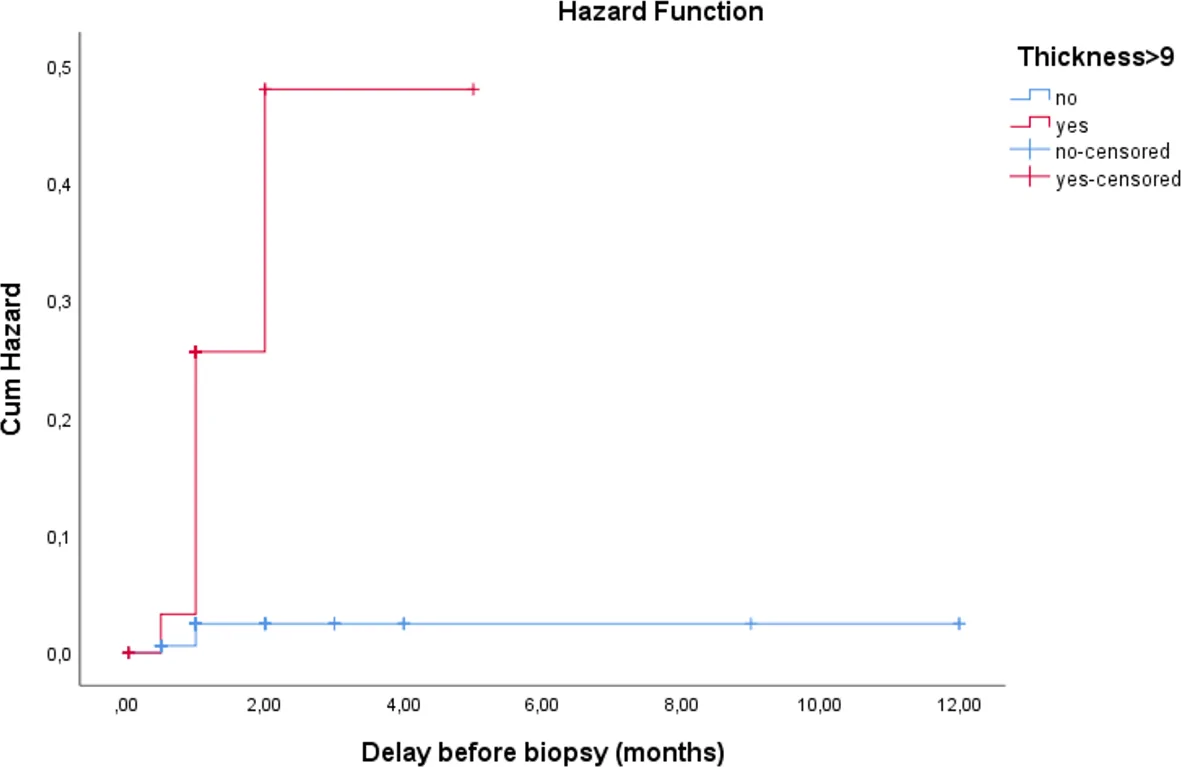
Kaplan–Meier survival analyses: time from initial consultation to definitive histological diagnosis (time-to-diagnosis).

Analysis of the interval from consultation to definitive histological diagnosis also revealed a significant difference (log-rank t χ ^2^ = 25.662, *p* < .0001): ( Figure 4).

- The median time-to-diagnosis was 12 months for the high-ET group, but not reached in the low-ET group, as fewer than 50% of patients in this group were diagnosed with malignancy.
- The mean time-to-diagnosis was shorter for the high-ET group (7.95 months, 95% CI: 4.74 – 11.16) compared to the low-ET group (11.73 months, 95% CI: 11.47 – 11.99).

This finding highlights the rapid clinical progression in the high-ET group compared to the indolent course associated with a thinner endometrium.

In summary, ET exceeding 9 mm is a critical marker that shortens both the patient-related delay in seeking care and the system-related delay in achieving a diagnosis. It effectively identifies a high-risk subgroup of premenopausal women with AUB who require urgent and efficient clinical evaluation.

## 4. Discussion

The evaluation of AUB in premenopausal women continues to represent a diagnostic dilemma. While ET measured by TVUS has been validated as a reliable screening tool in postmenopausal women, with a cutoff <4 mm effectively excluding endometrial cancer, ^4– 6^ its role in premenopausal women is far less clear.

Cyclical hormonal changes, structural abnormalities, and comorbidities contribute to marked variability in ET, limiting the establishment of universal thresholds. In this context, guidelines ^15, 16^ acknowledge the absence of consensus and recommend individualized evaluation based on risk factors and imaging findings.

### 4.1 Endometrial thickness cutoff in premenopausal AUB

Several studies have attempted to define an optimal cutoff value of ET for predicting endometrial pathology in women with AUB, yet the results remain inconsistent.

Our findings identify an ET >9 mm as the optimal threshold for predicting endometrial malignancy in premenopausal women.

This result aligns with earlier observations but provides more robust statistical validation.

Smith et al. ^17^ and Tongsong et al. ^18^ were among the first to suggest a correlation between thickened endometrium and pathology. Smith et al., ^17^ evaluating 51 premenopausal women with irregular bleeding, proposed an ET cutoff of 8 mm. In a larger study including 177 peri- and postmenopausal women undergoing vaginosonography, Tongsong et al. ^18^ found that an ET ≤7 mm reliably excluded endometrial pathology.

Subsequent studies yielded heterogeneous thresholds.

Getpook et al. ^19^ reported ≤8 mm as rarely malignant. Mayuri et al. ^20^ in a cross-sectional study of 62 perimenopausal women above 40 years of age, proposed an ET cutoff of ≥8 mm.

Luca et al., ^21^ in a cohort of 240 premenopausal women with AUB, identified >11 mm as the best cutoff for predicting endometrial carcinoma. This diagnostic performance was very close to Giannella et al. ^22^ who found ≥11 mm predictive in the presence of obesity or diabetes.

Ruan et al. ^23^ and Li et al. ^24^ incorporated ET ≥10 mm into multivariate prediction models, emphasizing its central role when combined with clinical variables.

Collectively, our findings bridge the gap between the lower cutoff values suggested in earlier work (7–8 mm) and the higher threshold of 11 mm reported by Luca et al., supporting >9 mm as a pragmatic and evidence-based threshold in premenopausal women with AUB.

### 4.2 Diagnostic performance of ET and AUC analysis

The diagnostic accuracy of ET in our cohort was high, with an AUC of 0.842. Sensitivity was 69.2% and specificity 87.1%, which is consistent with prior reports. ^20, 22^

Particularly noteworthy was the very high NPV (97.7%), highlighting the reliability of ET in excluding malignancy. This finding is in line with those of Smith et al. ^17^ and Mayuri et al., ^20^ who likewise reported NPVs exceeding 94%.

Consistent with our results, Kumari et al. ^25^ reported that a TVS-ET threshold of 10.5 mm achieved the highest diagnostic accuracy, with sensitivity and specificity of 89.5% and 86.3%, respectively, corresponding to an AUC of 0.920 (95% CI 0.846–0.994, p < 0.0001). Their cutoff also demonstrated a high NPV (95.7%), together with a PPV of 70.7% and favorable likelihood ratios, further supporting ET as a robust rule-out tool. ^24^

Conversely, the PPV was modest (26.5%), reflecting the frequent occurrence of benign causes of thickened endometrium such as polyps or hyperplasia. This aligns with the findings of Smith et al., ^17^ who reported an even lower PPV of 14%. Taken together with our results, this mirrors the broader literature, where ET is consistently shown to be an excellent rule-out tool but insufficient as a stand-alone diagnostic marker. ^19, 22, 23^

Nevertheless, by effectively excluding malignancy in low-risk patients, the cutoff has the potential to substantially reduce unnecessary endometrial biopsies and invasive interventions.

### 4.3 Integration with clinical risk factors

Although ET is highly informative, its predictive value increases when integrated with clinical parameters. Advanced age, obesity, diabetes, and hypertension are well-established contributors to endometrial carcinogenesis. ^23, 24^ For instance, Giannella et al. ^22^ demonstrated that the risk of cancer is significantly higher in obese women with ET ≥11 mm compared to their lean counterparts.

In our cohort, diabetes and hypertension were uncommon and not significantly associated with malignancy, likely reflecting the relatively small number of cancer cases (n = 13). Nevertheless, the combination of ET measurement with metabolic risk profiling remains promising, particularly in refining personalized risk stratification models.

In conclusion, the paramount importance of ET is its ability to stratify risk with high efficiency. It moves beyond mere association to provide actionable intelligence. By offering an objective, reproducible, and non-invasive metric, it forms the critical first step in a modern diagnostic algorithm for AUB, optimizing resource allocation, minimizing patient burden, and enhancing the overall precision of patient care.

### 4.4 Kaplan–Meier analysis: A novel prognostic perspective

A major innovation of our study lies in applying Kaplan–Meier survival analysis to ET, an approach not previously reported. It is worth reiterating that time-to-consultation is an indicator of clinical presentation dynamics, not of disease progression.

We demonstrated that women with ET >9 mm had significantly shorter time-to-consultation and time-to-diagnosis compared with those with ET ≤9 mm. This observation has two implications:

- First, it highlights ET as not only a static anatomical parameter but also a dynamic marker of disease burden: women with thicker endometrium likely experience more severe or symptomatic bleeding, prompting earlier medical attention.
- Second, it suggests a prognostic dimension, as ET may reflect both the biological aggressiveness of the lesion and its clinical expression.

Introducing Kaplan–Meier methodology into ET research thus provides a fresh lens for understanding patient trajectories and may inform prioritization strategies in healthcare systems with limited resources.

Our findings also have relevant implications for low-resource settings where access to hysteroscopy is limited or unavailable. In such contexts, the identification of an ET threshold >9 mm with high diagnostic accuracy (AUC 0.842) offers a practical and cost-effective triage strategy. ET measurement by TVUS, which is widely available and comparatively inexpensive, may help prioritize patients who require referral to higher-level facilities for further endometrial evaluation. This approach could optimize resource allocation, reduce unnecessary invasive procedures, and support earlier detection of malignant pathology in constrained healthcare systems.

### 4.5 Strengths and limitations

Our study has several notable strengths:

▪ **Relatively large cohort**: The study included 209 premenopausal women, making it one of the more substantial single-centre investigations of ET in the context of AUB.
▪ **Systematic evaluation of ET** by TVUS, following standardized IETA criteria, ensured methodological consistency and strengthened the internal validity of our findings.
▪ **Innovative introduction of survival analysis**: Which allowed us to explore not only diagnostic accuracy but also clinical dynamics such as time to consultation and time to diagnosis.
▪ **Use of definitive histopathology on hysterectomy specimens as the gold standard** minimized the risk of misclassification and reinforced diagnostic reliability.

However, some limitations must also be acknowledged:

▪ **Retrospective design** carries inherent risks of selection bias and incomplete data collection, with possible underreporting of confounders such as hormonal therapy use or lifestyle factors.
▪ **Absence of menstrual cycle phase standardization at the time of ultrasound:** Which is known to affect ET measurements and could have attenuated diagnostic performance.
▪ **Single-centre study conducted in a Tunisian tertiary hospital:** The findings may reflect specific referral patterns and population characteristics, thereby limiting generalizability.
▪ **External validation needed:** Although our proposed cutoff of >9 mm demonstrated excellent discriminative performance, it requires external validation in multicentric, prospective cohorts before it can be adopted in routine clinical practice.

### 4.6 Clinical implications

1. **Adoption of a >9 mm cutoff**: ET >9 mm can be used as a pragmatic and evidence-based threshold for risk stratification in premenopausal women with AUB.
2. **Conservative management in low-risk patients**: For women with ET ≤9 mm, the probability of malignancy is extremely low. This supports a conservative, non-invasive management strategy and reduces reliance on unnecessary procedures such as hysteroscopy or curettage.
3. **Trigger for histological assessment**: ET >9 mm with premenopausal AUB should prompt immediate histological sampling, even if other clinical risk factors (e.g., obesity, diabetes, family history) are absent. This ensures early detection and prevents diagnostic delay.
4. **Guidance for referral and timing of intervention**: Kaplan–Meier findings demonstrate that women with ET >9 mm tend to present earlier and reach diagnosis faster. ET can thus serve as a practical tool for prioritizing referrals and expediting intervention in high-risk cases.
5. **Dual diagnostic and prognostic role**: ET functions not only as a diagnostic marker but also as a potential surrogate marker reflecting disease burden. This dual role reinforces its central place in the clinical evaluation of premenopausal AUB.

### 4.7 Recommendations for further research

1. **Prospective validation of the >9 mm cutoff**: Our results support ET >9 mm as a reliable threshold for ruling out malignancy in premenopausal women with AUB. However, prospective, multicenter studies are needed to confirm its robustness across diverse clinical settings, patient populations, and ultrasound operators.
  Such validation would enhance external generalizability and facilitate adoption into clinical guidelines.
2. **Cross-ethnic and cross-geographic evaluation**: Endometrial pathology prevalence, risk factors, and clinical presentation may differ according to ethnicity, genetics, lifestyle, and healthcare access. Studies in varied regions and populations are essential to determine whether the >9 mm cutoff is universally applicable or whether ethnicity-specific thresholds are warranted.
3. **Integration into multivariate risk models**: ET alone, while powerful, has limited PPV. Future research should investigate the incorporation of ET into multifactorial models that also include age, BMI, metabolic status (diabetes, hypertension), hormonal profiles, and molecular biomarkers. Such models may outperform ET alone, enabling more precise and individualized risk stratification.
4. **Expansion of Kaplan–Meier methodology**: Our study is the first to apply Kaplan–Meier analysis to explore diagnostic timelines in AUB. Further research should extend this approach to assess long-term outcomes, such as recurrence, progression from hyperplasia to carcinoma, and survival rates. This would position ET not only as a diagnostic triage tool but also as a prognostic marker of disease trajectory.
5. **Evaluation of cost-effectiveness and patient-centered outcomes**: Beyond diagnostic accuracy, studies should assess the economic implications of using ET as a triage criterion, particularly in resource-limited settings. Research should also explore patient-reported outcomes, including anxiety reduction, satisfaction, and quality of life when invasive procedures are avoided based on reassuring ET results.

## 5. Conclusions

This study reinforces the pivotal role of ET as a key non-invasive parameter in the evaluation of premenopausal women with AUB. We identified an ET >9 mm as an independent predictor of malignancy, with excellent NPV, supporting its use as a triage threshold to reduce unnecessary invasive procedures. Beyond its diagnostic accuracy, our application of Kaplan–Meier survival analysis provides an innovative perspective, suggesting that ET may also reflect disease dynamics and influence the timing of clinical presentation.

Our findings add to growing evidence that integrating ET with clinical risk factors can enhance predictive accuracy, and that simplified risk scores may outperform ET alone in guiding selective endometrial sampling.

While external validation is needed, this study provides a clinically relevant framework that could improve patient stratification, optimize use of diagnostic resources, and ultimately contribute to earlier and more targeted detection of endometrial malignancy in women with AUB.

## Ethical considerations

We confirm adherence to the Journal’s ethical publication standards. The study protocol was approved by the Institutional Ethics Committee of Charles Nicolle Hospital, Tunis, Tunisia, on March 6, 2025 (approval number: FWA 00032748–IORG0011243).

## Consent to participate

As this was a retrospective study using anonymized data, the requirement for informed consent was waived.

## Data Availability

All data sets can be assessed and all study findings reported in the article are shared via Harvard Dataverse: “Endometrial Thickness as a Prognostic Marker in Premenopausal Abnormal Uterine Bleeding: Diagnostic Accuracy and Kaplan–Meier Survival Insights,” https://doi.org/10.7910/DVN/W6QVWI. ^26^ This project contains the following:
• Dataset Endometrial Thickness • Study findings (1) • Study findings (2) Harvard Dataverse: “Endometrial Thickness as a Prognostic Marker in Premenopausal Abnormal Uterine Bleeding: Diagnostic Accuracy and Kaplan–Meier Survival Insights,” https://doi.org/10.7910/DVN/W6QVWI. ^26^ This project contains the following:
• Canvas (English) This work has been reported in line with the STROBE guidelines. ^27^ Harvard Dataverse: “Endometrial Thickness as a Prognostic Marker in Premenopausal Abnormal Uterine Bleeding: Diagnostic Accuracy and Kaplan–Meier Survival Insights,” https://doi.org/10.7910/DVN/W6QVWI. ^26^ This project contains the following:
• STROBE Checklist Data are available under the terms of the Creative Commons Zero “No rights reserved” data waiver (CC0 1.0 Public domain dedication).
