# Supplementary material for: Endometrial Thickness as a Prognostic Marker in Premenopausal Abnormal Uterine Bleeding: Diagnostic Accuracy and Kaplan–Meier Survival Insights
Source: F1000Res. 2026 May 19;14:1167. Originally published 2025 Oct 27. [Version 4] doi: 10.12688/f1000research.170554.4 (PMC13560006; doi:10.12688/f1000research.170554.4)
Supplement: Peer review history

# Reviewer report: Reviewer response for version 4

**DOI:** 10.5256/f1000research.200673.r486405

**Published:** 2026-08-26

**Authors:** Deepthy Balakrishnan

**Affiliations:** 1 All India Institute of Medical Sciences, Odisha, India

The revised text addresses the queries; however, the results should be interpreted with caution because menstrual cycle phases are not standardised.

Is the work clearly and accurately presented and does it cite the current literature?

Yes

If applicable, is the statistical analysis and its interpretation appropriate?

Yes

Are all the source data underlying the results available to ensure full reproducibility?

Yes

Is the study design appropriate and is the work technically sound?

Yes

Are the conclusions drawn adequately supported by the results?

Yes

Are sufficient details of methods and analysis provided to allow replication by others?

Yes

Reviewer Expertise:

obstetrics, gynecology, maternal medicine,

I confirm that I have read this submission and believe that I have an appropriate level of expertise to confirm that it is of an acceptable scientific standard.

# Reviewer report: Reviewer response for version 4

**DOI:** 10.5256/f1000research.200673.r486406

**Published:** 2026-06-24

**Authors:** CANSU TURKER SARICOBAN

**Affiliations:** 1 Pathology, Istanbul University-Cerrahpasa, Cerrahpasa Faculty of Medicine, Istanbul, Turkey

Thank you for the detailed response and for the substantial revisions made to the manuscript. Several concerns have been addressed appropriately, including clarification of the retrospective design, the definition of premenopausal women, the ultrasound measurement procedure, the number and experience of operators, the lack of menstrual cycle phase standardization, the absence of formal interobserver agreement analysis, and the concordance between preoperative sampling and hysterectomy specimens.

 However, important concerns remain.

**1. Prognostic interpretation remains unsupported**

 The response letter correctly acknowledges that time-to-consultation primarily reflects healthcare-seeking behavior rather than biological disease progression. However, the manuscript still describes ET as a prognostic marker and continues to use expressions such as “aggressive clinical course,” “rapid clinical progression,” “indolent course,” “biological aggressiveness,” and “dual diagnostic and prognostic role.”

 The present study does not assess established prognostic outcomes such as stage, grade, depth of invasion, recurrence, progression, disease-specific survival, or overall survival. Therefore, ET may be discussed as a diagnostic triage or risk-stratification parameter, but not as a prognostic marker of biological aggressiveness.

**2. Kaplan–Meier analyses remain unclear**

 Kaplan–Meier analysis may be used for time-to-event outcomes, but the event definitions and censoring rules are not sufficiently explained.

 For time-to-diagnosis, the manuscript states that the median was not reached in the low-ET group because fewer than 50% of patients were diagnosed with malignancy. This suggests that the event may have been defined as diagnosis of malignancy rather than definitive histological diagnosis. If so, the analysis should not be interpreted as diagnostic delay.

 The figures also appear to be labelled as hazard functions rather than Kaplan–Meier survival curves, and the identical log-rank chi-square value reported for both analyses should be verified.

 The Kaplan–Meier section should either be removed or fully reframed as an exploratory assessment of diagnostic pathways, without prognostic interpretation.

**3. The >9 mm threshold is clinically overstated**

 Among patients with ET ≤9 mm, four malignancies were identified. The reported sensitivity is only 69.2%, meaning that nearly one-third of malignant cases would be missed if ET were used alone.

 Therefore, the data do not support statements that ET ≤9 mm safely rules out malignancy or justifies conservative management without histological assessment. The high negative predictive value should also be interpreted cautiously because it is prevalence-dependent and derived from a selected cohort of women who underwent hysterectomy.

 ET should be presented as one component of a multifactorial risk assessment, not as a stand-alone gatekeeper.

**4. Selection bias and model instability remain important limitations**

 The study includes only women who underwent hysterectomy, which limits generalizability to the broader population of premenopausal women with AUB.

 In addition, only 13 malignant cases were identified, yet six predictors were entered into the multivariable model. The very wide confidence intervals indicate model instability and probable overfitting. These results should be described as exploratory rather than robust independent predictors.

**5. Reporting inconsistencies persist**

 The percentages reported for family cancer history, diabetes, and hypertension remain difficult to interpret and appear inconsistent with the corresponding p values. Table 2 also contains misaligned or inconsistent categories. These should be carefully revised.

 Minor linguistic and typographical errors also remain, including phrases such as “on 6 March 2025 by before conducting the study” and “Tandardization.”

**Conclusion**

 The manuscript has improved substantially, but the central interpretative issue remains unresolved. The study may support an exploratory diagnostic or risk-stratification role for ET, but it does not demonstrate prognostic value or biological aggressiveness.

 For these reasons, I do not consider the manuscript suitable for approval in its current form.

Is the work clearly and accurately presented and does it cite the current literature?

Partly

If applicable, is the statistical analysis and its interpretation appropriate?

Partly

Are all the source data underlying the results available to ensure full reproducibility?

Partly

Is the study design appropriate and is the work technically sound?

Partly

Are the conclusions drawn adequately supported by the results?

No

Are sufficient details of methods and analysis provided to allow replication by others?

Partly

Reviewer Expertise:

Gynaecopathology

I confirm that I have read this submission and believe that I have an appropriate level of expertise to state that I do not consider it to be of an acceptable scientific standard, for reasons outlined above.

# Reviewer report: Reviewer response for version 3

**DOI:** 10.5256/f1000research.194905.r475875

**Published:** 2026-04-27

**Authors:** Deepthy Balakrishnan

**Affiliations:** 1 All India Institute of Medical Sciences, Odisha, India

1. It is a retrospective observational study

 2.  Measurement of the details of ET is not specified for any particular phase of the menstrual cycle, but this has been mentioned in the limitations. Nevertheless, it creates a bias.

 3. Please clarify the definition of "premenopausal women"

 4. 4. Tamoxifen should come under the exclusion criteria because of the differences in ET caused by subepithelial stromal hypertrophy

 5. It would have been better to have a combined score for risk stratification rather than an isolated ET because of various confounders 

 The study aims to determine a cut-off value for endometrial thickness in premenopausal women with AUB. There is a difference in ET across various phases of the cycle, and it may also vary in women already on hormones to control bleeding. These can cause profound changes in the results.

 The article is beautifully written with good statistical analysis. The confounders may reduce the reproducibility of the data with difficulty in incorporating in to medical practice

Is the work clearly and accurately presented and does it cite the current literature?

Yes

If applicable, is the statistical analysis and its interpretation appropriate?

Yes

Are all the source data underlying the results available to ensure full reproducibility?

Yes

Is the study design appropriate and is the work technically sound?

Yes

Are the conclusions drawn adequately supported by the results?

Yes

Are sufficient details of methods and analysis provided to allow replication by others?

Yes

Reviewer Expertise:

obstetrics, gynecology, maternal medicine,

I confirm that I have read this submission and believe that I have an appropriate level of expertise to confirm that it is of an acceptable scientific standard, however I have significant reservations, as outlined above.

# Response

**Published:** 2026-05-04

**Authors:** Narjes karmous

**Affiliations:** University of Tunis El Manar Faculty of Medicine of Tunis, Tunis, Tunis, Tunisia

Dear Dr. Deepthy Balakrishnan,

 We sincerely thank you for your thoughtful and constructive feedback on our manuscript. We greatly appreciate your positive assessment of the clarity of the manuscript, the statistical analysis and the overall scientific presentation. We have carefully considered all the points raised and revised the manuscript accordingly. Our detailed responses and the corresponding modifications are presented below.

**1. “It is a retrospective observational study.”**

**Response:**

 Thank you for this important observation. We agree that the study design should be described using standard epidemiological terminology. Accordingly, we revised the manuscript to describe the study as a retrospective observational study. The terminology was corrected in both the Abstract and the Methods section.

**2. “Measurement of the details of ET is not specified for any particular phase of the menstrual cycle, but this has been mentioned in the limitations. Nevertheless, it creates a bias.”**

**Response:**

 We fully agree that the absence of menstrual cycle phase standardization represents an important source of bias in the interpretation of endometrial thickness (ET) in premenopausal women.

 Because of the retrospective design of the study and the high frequency of irregular, anovulatory, or unpredictable bleeding patterns among women presenting with abnormal uterine bleeding (AUB), menstrual cycle phase at the time of ultrasound examination was not consistently documented in medical records and therefore could not be standardized retrospectively.

 Our intention was not to establish a physiological cycle-specific ET reference value, but rather to evaluate ET as a pragmatic clinical marker in real-world symptomatic premenopausal women with AUB. To avoid overinterpretation, we further clarified in the manuscript that the proposed ET cutoff should be interpreted as a risk-stratification threshold within a clinical context rather than as a universal physiological cutoff.

 Additional clarifications were added to the Methods and Limitations sections regarding the absence of menstrual cycle phase standardization and its potential impact on ET interpretation. We emphasized that prospective studies incorporating cycle-phase standardization are necessary to improve reproducibility and biological interpretability.

**3. “Please clarify the definition of premenopausal women.”**

**Response:**

 Thank you for highlighting the need for a clearer definition. We have now explicitly defined premenopausal women in the Methods section. The following definition was added: “Premenopausal women were defined as women with ongoing menstrual activity who had not experienced 12 consecutive months of amenorrhea unrelated to pregnancy, lactation or hormonal therapy.”

**4. “Tamoxifen should come under the exclusion criteria because of the differences in ET caused by subepithelial stromal hypertrophy.”**

**Response:**

 Thank you for this important comment. We agree that tamoxifen exposure may alter endometrial morphology and thickness, potentially affecting the interpretation of ET.

 However, in our cohort, only one patient had a history of tamoxifen use. Given this extremely limited exposure, we believe that tamoxifen was unlikely to have had a significant impact on the overall results or on the proposed ET cutoff value.

 Therefore, no additional exclusion criterion was introduced retrospectively, as excluding this single patient would not have materially altered the study findings.

 We nevertheless acknowledge that tamoxifen exposure may represent a potential confounding factor in larger cohorts evaluating ET in premenopausal women with abnormal uterine bleeding.

**5. “It would have been better to have a combined score for risk stratification rather than an isolated ET because of various confounders.”**

**Response:**

 Thank you for this valuable suggestion. We fully agree that ET alone cannot entirely capture the complexity of endometrial malignancy risk in premenopausal women with AUB, particularly given the influence of multiple clinical and hormonal confounding factors.

 The primary objective of the present study was to specifically evaluate the diagnostic performance of ET as a simple, non-invasive and widely accessible imaging marker in routine clinical practice. However, we agree that integrating ET into multifactorial risk prediction models combining clinical, metabolic, hormonal and imaging variables may improve predictive accuracy and clinical applicability.

 This perspective has been emphasized in the Recommendations for further research section.

 We sincerely thank you again for your insightful comments and constructive suggestions. Your observations significantly contributed to improving the methodological transparency, scientific rigor and clinical interpretation of our manuscript.

 Kind regards,

 Narjes Karmous

 On behalf of all authors

# Reviewer report: Reviewer response for version 3

**DOI:** 10.5256/f1000research.194905.r448964

**Published:** 2026-01-12

**Authors:** CANSU TURKER SARICOBAN

**Affiliations:** 1 Pathology, Istanbul University-Cerrahpasa, Cerrahpasa Faculty of Medicine, Istanbul, Turkey

 I thank the authors for their revisions and for addressing several points raised by the other reviewer. However, after carefully reviewing Version 2 of the manuscript, I find that the major methodological concerns raised in my original review remain largely unaddressed.

 In particular, the absence of menstrual cycle phase standardization for endometrial thickness measurement continues to fundamentally limit the biological and diagnostic interpretability of the proposed cutoff. Reframing this issue as a “real-world” approach does not resolve the underlying methodological problem, but rather highlights it.

 Similarly, the restriction of the study population to patients who ultimately underwent hysterectomy introduces substantial selection bias, which cannot be adequately mitigated by post hoc clarification. The use of Kaplan–Meier analysis in this context, even when reinterpreted as reflecting healthcare-seeking behavior rather than prognosis, remains conceptually problematic and unsupported by the study design.

 Finally, the low number of malignant events continues to undermine the robustness of the ROC analysis and time-to-event modeling. While these limitations are now more explicitly acknowledged, acknowledgment alone does not sufficiently address their impact on the validity of the study’s conclusions.

 For these reasons, I maintain my original assessment that the manuscript does not meet acceptable methodological standards for approval.

Is the work clearly and accurately presented and does it cite the current literature?

Partly

If applicable, is the statistical analysis and its interpretation appropriate?

Partly

Are all the source data underlying the results available to ensure full reproducibility?

Partly

Is the study design appropriate and is the work technically sound?

Partly

Are the conclusions drawn adequately supported by the results?

No

Are sufficient details of methods and analysis provided to allow replication by others?

Partly

Reviewer Expertise:

Gynaecopathology

I confirm that I have read this submission and believe that I have an appropriate level of expertise to state that I do not consider it to be of an acceptable scientific standard, for reasons outlined above.

# Reviewer report: Reviewer response for version 3

**DOI:** 10.5256/f1000research.194905.r448963

**Published:** 2026-01-10

**Authors:** Seetu Palo

**Affiliations:** 1 All India Institute of Medical Sciences, Hyderabad, India

Revisions made are satisfactory.

Is the work clearly and accurately presented and does it cite the current literature?

Partly

If applicable, is the statistical analysis and its interpretation appropriate?

Partly

Are all the source data underlying the results available to ensure full reproducibility?

Partly

Is the study design appropriate and is the work technically sound?

Partly

Are the conclusions drawn adequately supported by the results?

No

Are sufficient details of methods and analysis provided to allow replication by others?

Partly

Reviewer Expertise:

Oncopathology

I confirm that I have read this submission and believe that I have an appropriate level of expertise to confirm that it is of an acceptable scientific standard.

# Reviewer report: Reviewer response for version 1

**DOI:** 10.5256/f1000research.188024.r428684

**Published:** 2025-11-20

**Authors:** CANSU TURKER SARICOBAN

**Affiliations:** 1 Pathology, Istanbul University-Cerrahpasa, Cerrahpasa Faculty of Medicine, Istanbul, Turkey

The authors present a retrospective analytical study evaluating the diagnostic and purported prognostic utility of transvaginal ultrasound–based endometrial thickness (ET) in premenopausal women with abnormal uterine bleeding (AUB). The study aims to define an optimal ET cutoff (>9 mm) for predicting endometrial malignancy and further applies Kaplan–Meier time-to-event analyses (time-to-consultation and time-to-diagnosis) to explore clinical dynamics associated with different ET thresholds. Although the topic is clinically relevant and the dataset includes hysterectomy-confirmed outcomes, the study suffers from substantial methodological, analytical, and reporting limitations that significantly compromise the validity, reliability, and generalizability of its findings.

 A fundamental methodological limitation is the complete lack of menstrual cycle phase standardization at the time of ET measurement. Endometrial thickness varies markedly between the proliferative and secretory phases, making ET uninterpretable without cycle context. This single issue undermines the scientific foundation of the proposed cutoff value. Additionally, ET assessment via transvaginal ultrasound is known to have considerable interobserver variability; yet the manuscript does not state whether measurements were performed by a single operator or multiple clinicians, nor does it report any interobserver agreement or standardization procedures. These omissions severely restrict reproducibility and weaken the internal validity of the imaging data.

 The decision to use hysterectomy specimens as the “gold standard” for definitive diagnosis is also problematic. In a subset of endometrial carcinomas—particularly early or superficially located tumors—the entire neoplastic tissue may be removed by curettage or hysteroscopy, leading to an absent residual tumor in the hysterectomy specimen. The authors do not report any comparison between preoperative biopsy/hysteroscopy findings and post-hysterectomy pathology, nor do they address the risk of misclassification bias arising from these discrepancies. This omission raises concerns about reference standard accuracy.

 Furthermore, the presentation of baseline characteristics is confusing and potentially misleading. Percentages reported for variables such as diabetes, hypertension, and family history (“93% benign vs. 7% malignant”) are not clearly defined—whether they refer to patients with the risk factor, patients without it, or proportions within total groups is unclear. This ambiguity compromises interpretation of the risk factor analysis and represents a deviation from appropriate epidemiologic reporting standards.

 There are also inconsistencies within the dataset itself. For example, the manuscript states that the median ET in malignant cases is 12 mm, whereas Table 1 appears to present a different value (e.g., 10 mm or an incomplete notation). Such discrepancies reduce confidence in the accuracy and integrity of the reported data. Additionally, the application and interpretation of Kaplan–Meier analysis are conceptually flawed. “Time-to-consultation” reflects health-seeking behavior rather than biological disease progression and should not be presented as a prognostic marker. Given that symptom onset times were extracted retrospectively, this metric is particularly vulnerable to recall bias. The malignant event count (n=13) is also extremely low, limiting the statistical power of ROC analyses, logistic regression, and survival models, and rendering the resulting cutoff and effect estimates unstable.

 Lastly, the sampling frame introduces significant selection bias: only women who ultimately underwent hysterectomy were included. This subgroup represents a higher-risk and highly selected population that does not reflect the broader premenopausal AUB cohort seen in routine practice. Consequently, the >9 mm cutoff cannot be generalized beyond this specific surgical population.

 The manuscript contains several typographical and formatting errors (e.g., “8- year and 11-month,” “Mars 2025”), and the tone of certain phrases (“cornerstone parameter,” “robust prognostic marker”) is overstated given the study’s methodological limitations. Table formatting requires substantial correction, as percentages, p-values, and variable categories are inconsistently aligned or inadequately defined. Some terminology is unclear or unconventional (e.g., “intracavitary fibroma”), and adherence to STROBE reporting guidelines is incomplete.

 While the research topic is important, the methodological flaws, analytical limitations, inconsistent reporting, and conceptual misinterpretations present in the current version significantly compromise the validity of the findings. Extensive revisions, additional clarifications, and methodological restructuring would be required before this work could be considered for indexing. As it stands, the manuscript is not acceptable in its current form.

Is the work clearly and accurately presented and does it cite the current literature?

Partly

If applicable, is the statistical analysis and its interpretation appropriate?

Partly

Are all the source data underlying the results available to ensure full reproducibility?

Partly

Is the study design appropriate and is the work technically sound?

Partly

Are the conclusions drawn adequately supported by the results?

No

Are sufficient details of methods and analysis provided to allow replication by others?

Partly

Reviewer Expertise:

Gynaecopathology

I confirm that I have read this submission and believe that I have an appropriate level of expertise to state that I do not consider it to be of an acceptable scientific standard, for reasons outlined above.

# Response

**Published:** 2026-01-06

**Authors:** Narjes karmous

**Affiliations:** University of Tunis El Manar Faculty of Medicine of Tunis, Tunis, Tunis, Tunisia

Dear dr CANSU TURKER SARICOBAN,

 We sincerely thank you for your thorough and constructive feedback on our manuscript. We have carefully addressed all the points raised. Our responses and the corresponding changes made in the manuscript are summarized below:

***1. A fundamental methodological limitation is the complete lack of menstrual cycle phase standardization at the time of ET measurement. ET varies markedly between the proliferative and secretory phases, making ET uninterpretable without cycle context. This single issue undermines the scientific foundation of the proposed cutoff value.***

**Response:**

 Thank you for raising this important methodological concern. We fully agree that endometrial thickness (ET) varies according to the menstrual cycle phase and that phase-specific measurement represents the optimal physiological approach.

 However, the present study was designed to reflect routine clinical practice in premenopausal women presenting with abnormal uterine bleeding (AUB), a population in whom menstrual cycle regularity and accurate phase determination are frequently unavailable or unreliable at the time of clinical assessment. Consequently, ET measurements were performed at presentation, irrespective of cycle phase, in line with real-world diagnostic pathways.

 Our objective was therefore not to define a physiological, cycle-dependent reference value for ET, but rather to evaluate its diagnostic and prognostic utility as a pragmatic clinical marker for identifying women at increased risk of endometrial malignancy in a symptomatic AUB population. Importantly, several previously published retrospective studies addressing ET in premenopausal AUB populations similarly lacked systematic cycle phase standardization and nonetheless reported clinically useful cutoff values.

 We acknowledge that the absence of menstrual cycle phase standardization represents a major limitation of our study and have explicitly addressed this in the Methods and Limitations sections. To avoid overinterpretation, we have also clarified that the proposed ET cutoff should be interpreted as a risk stratification threshold within a clinical context, rather than as an absolute physiological value. We agree that future prospective studies incorporating precise cycle phase control are required to refine and validate phase-specific thresholds.

***2. The manuscript does not state whether measurements were performed by a single operator or multiple clinicians, nor does it report any interobserver agreement or standardization procedures. These omissions severely restrict reproducibility and weaken the internal validity of the imaging data.***

**Response:**

 Thank you for highlighting this important aspect related to reproducibility and internal validity. We have revised the Methods section to clarify that all TVUS examinations were performed by three experienced operators, each holding an inter-university diploma in gynecologic US and with over 10 years of clinical experience in pelvic US practice.

 Given the retrospective nature of the study, formal assessment of interobserver agreement (e.g., intraclass correlation coefficients) and predefined standardization protocols could not be performed. However, all operators followed institutional US protocols for endometrial assessment, and ET was measure in the sagittal plane as the double-layer distance between the two endometrial–myometrial interfaces, at the point of greatest thickness perpendicular to the midline, using appropriately magnified images, in accordance with standard ultrasound assessment protocols used at our institution.

 We acknowledge that the absence of formal interobserver variability analysis represents a limitation and have explicitly addressed this in the Limitations section. Future prospective studies incorporating predefined measurement protocols and interobserver agreement assessment are warranted to further strengthen reproducibility.

**3. The decision to use hysterectomy specimens as the “gold standard” for definitive diagnosis is also problematic. In a subset of endometrial carcinomas—particularly early or superficially located tumors—the entire neoplastic tissue may be removed by curettage or hysteroscopy, leading to an absent residual tumor in the hysterectomy specimen. The authors do not report any comparison between preoperative biopsy/hysteroscopy findings and post-hysterectomy pathology, nor do they address the risk of misclassification bias arising from these discrepancies. This omission raises concerns about reference standard accuracy.**

**Response:**

 Thank you for raising this important point regarding the use of hysterectomy specimens as the reference standard. We agree that in some series, early or superficially located endometrial tumors may be completely removed during curettage or hysteroscopy, potentially leading to absent residual tumor in the hysterectomy specimen and misclassification. In our study, we systematically compared preoperative endometrial biopsy or hysteroscopy findings with post-hysterectomy pathology. This comparison demonstrated excellent **concordance**, with no discrepancies observed. This result has now been added to the **Results **section to clarify that hysterectomy specimens provided a reliable reference standard in our cohort.

**4. Percentages reported for variables such as diabetes, hypertension, and family history (“93% benign vs. 7% malignant”) are not clearly defined—whether they refer to patients with the risk factor, patients without it, or proportions within total groups is unclear. This ambiguity compromises interpretation of the risk factor analysis and represents a deviation from appropriate epidemiologic reporting standards.**

**Response:**

 We have revised the Results section to report the presence of comorbidities rather than their absence, in accordance with epidemiological reporting standards. Percentages now clearly represent the proportion of patients presenting each risk factor within the benign and malignant groups, eliminating any ambiguity.

**5. The manuscript states that the median ET in malignant cases is 12 mm, whereas Table 1 appears to present a different value (e.g., 10 mm or an incomplete notation). Such discrepancies reduce confidence in the accuracy and integrity of the reported data.**

**Response:**

 Thank you for drawing our attention to this discrepancy. We confirm that the **correct median ET in malignant cases is 12 mm** **.** The value initially reported in **Table 1** (10 mm) resulted from a **data transcription error** and has now been **corrected** **.**

 In addition, following the request of Reviewer 1, a new table was added to the Methods section, and the baseline characteristics table has therefore been **renumbered as Table 2** **.** The corrected ET value is now consistently reported in both the Results text and **Table 2** **.**

 All numerical values reported in the text and tables were cross-checked to ensure internal consistency.

**6. The application and interpretation of Kaplan–Meier analysis are conceptually flawed. “Time-to-consultation” reflects health-seeking behavior rather than biological disease progression and should not be presented as a prognostic marker. Given that symptom onset times were extracted retrospectively, this metric is particularly vulnerable to recall bias.**

**Response:**

 We agree that “ *time-to-consultation”* primarily reflects health-seeking behavior rather than intrinsic biological disease progression and, as such, should not be interpreted as a biological prognostic marker. In the revised manuscript, we have therefore **clarified the objective and interpretation** of the Kaplan–Meier analysis. The analysis is now explicitly presented as an **exploratory assessment of diagnostic delay**, illustrating differences in healthcare-seeking patterns according to endometrial thickness, rather than as a predictor of tumor aggressiveness or prognosis.

 We also acknowledge that symptom onset was collected retrospectively and is therefore susceptible to **recall bias** **.** This limitation has now been clearly stated in the **Discussion/Limitations** section. Importantly, the Kaplan–Meier analysis is no longer framed as prognostic, and its results are interpreted with appropriate caution.

**7. The malignant event count (n=13) is also extremely low, limiting the statistical power of ROC analyses, logistic regression, and survival models, and rendering the resulting cutoff and effect estimates unstable.**

**Response:**

 We acknowledge that the low number of malignant events (n = 13) limits the statistical power of the ROC analyses, logistic regression, and Kaplan–Meier models, and may result in unstable cutoff values and imprecise effect estimates.

 This limitation has now been explicitly stated in the **Discussion/Limitations** section, and the results of these analyses are interpreted with appropriate caution. We emphasize that our findings should be considered exploratory and hypothesis-generating rather than definitive.

***8. Several typographical and formatting errors (e.g., “8- year and 11-month,” “Mars 2025”), and the tone of certain phrases (“cornerstone parameter,” “robust prognostic marker”) is overstated given the study’s methodological limitations. Table formatting requires substantial correction, as percentages, p-values, and variable categories are inconsistently aligned or inadequately defined. Some terminology is unclear or unconventional (e.g., “intracavitary fibroma”), and adherence to STROBE reporting guidelines is incomplete.***

**Response:**

 We have carefully revised the manuscript to address all typographical, stylistic, and formatting issues identified.

 All typographical and linguistic errors have been corrected, including inconsistencies in date and duration formatting (e.g., “8-year and 11-month period,” “March 2025”). The tone of the manuscript has been moderated to better reflect the methodological limitations of the study, and overstated expressions such as “cornerstone parameter” and “robust prognostic marker” have been replaced with more cautious and precise terminology.

 All tables have been comprehensively reformatted to ensure consistent alignment of variables, percentages, and p-values, with clear definition of categories and denominators where applicable. Terminology has been standardized in accordance with internationally accepted nomenclature, and ambiguous terms such as “intracavitary fibroma” have been replaced with clearer expressions (e.g., “intracavitary fibroid”).

 Finally, we have reviewed the manuscript in accordance with the STROBE reporting guidelines for observational studies and have ensured that all relevant items are adequately addressed. The manuscript sections have been revised to improve transparency and reporting completeness.

# Reviewer report: Reviewer response for version 1

**DOI:** 10.5256/f1000research.188024.r428689

**Published:** 2025-11-06

**Authors:** Seetu Palo

**Affiliations:** 1 All India Institute of Medical Sciences, Hyderabad, India

This article presents a retrospective analytical study investigating the diagnostic and prognostic value of endometrial thickness (ET) in premenopausal women with abnormal uterine bleeding (AUB). The authors aim to define an optimal ET cutoff for predicting endometrial malignancy and to explore the prognostic implications of ET using Kaplan–Meier survival analysis. The manuscript is generally well-written and addresses a clinically relevant topic, but certain aspects require clarification, additional rigor, and scientific refinement.

 In the abstract, clearly clarify that Kaplan–Meier analysis was applied to time-to-consultation and time-to-diagnosis, not survival outcomes. Include the AUC value (0.842) and the >9 mm cutoff directly in the abstract. The concept of “time-to-consultation” as a survival endpoint is unorthodox. It reflects healthcare-seeking behavior rather than biological survival. Consider reframe this as an auxiliary behavioural metric rather than a true prognostic parameter. Revise “prognostic marker” to “potential prognostic or triage marker.”

 In the methodology section, please specify whether TVUS was performed by a single operator or multiple radiologists. In section 2.4, consider including details of variables retained in the multivariate model and present odds ratios in a table. Similarly in the results, consider providing a regression summary table (with adjusted ORs and 95% CIs). Furthermore, Table 1 formatting can be improved (alignment of percentages and p-values). In the discussion section, clarify that “time-to-consultation” is an indicator of clinical presentation dynamics, not disease progression. A brief discussion on the implications for low-resource settings where hysteroscopy access is limited, can be added. Rather than claiming ET as a “robust prognostic marker,” the phrase “a potential surrogate marker reflecting disease burden” is suggested. Similarly, the phrase “cornerstone parameter” may be moderated to “key non-invasive parameter.” Formatting and minor errors also needs rectification, such as, correct “8- year and 11-month” to “8 years and 11 months” and “On 6 Mars 2025” to “on March 6, 2025.”

Is the work clearly and accurately presented and does it cite the current literature?

Partly

If applicable, is the statistical analysis and its interpretation appropriate?

Partly

Are all the source data underlying the results available to ensure full reproducibility?

Partly

Is the study design appropriate and is the work technically sound?

Partly

Are the conclusions drawn adequately supported by the results?

No

Are sufficient details of methods and analysis provided to allow replication by others?

Partly

Reviewer Expertise:

Oncopathology

I confirm that I have read this submission and believe that I have an appropriate level of expertise to state that I do not consider it to be of an acceptable scientific standard, for reasons outlined above.

# Response

**Published:** 2025-11-15

**Authors:** Narjes karmous

**Affiliations:** University of Tunis El Manar Faculty of Medicine of Tunis, Tunis, Tunis, Tunisia

Dear dr Seetu Palo,

 We sincerely thank you for your thorough and constructive feedback on our manuscript. We have carefully addressed all the points raised. Our responses and the corresponding changes made in the manuscript are summarized below:

**1. Abstract – Kaplan–Meier analysis, AUC, cutoff, and terminology**

**Reviewer comment:**

 Clearly clarify that Kaplan–Meier analysis was applied to time-to-consultation and time-to-diagnosis, not survival outcomes. Include the AUC value (0.842) and the >9 mm cutoff directly in the abstract. The concept of “time-to-consultation” as a survival endpoint is unorthodox. Consider reframe this as an auxiliary behavioural metric rather than a true prognostic parameter. Revise “prognostic marker” to “potential prognostic or triage marker.”

**Response:**

 We have revised the abstract accordingly.

- Kaplan–Meier analysis is now clearly described as applied to **time-to-consultation and time-to-diagnosis**, reflecting healthcare-seeking behavior rather than survival.
- The **AUC value (0.842)** and the **>9 mm cutoff** are explicitly included.
- Terminology “robust prognostic marker” has been replaced with **“potential prognostic or triage marker”**.
- The concept of time-to-consultation is framed as an **auxiliary behavioral metric**, not a biological survival outcome.

**2. Methodology – TVUS operators and multivariate model**

**Reviewer comment:**

 Please specify whether TVUS was performed by a single operator or multiple radiologists. In section 2.4, consider including details of variables retained in the multivariate model and present odds ratios in a table.

**Response:**

- We have added in the Methods section (2.2 and 2.4) that **three qualified operators with inter-university diplomas in ultrasound and at least 10 years of experience** performed all TVUS examinations.
- In section 2.4, we have specified the **variables included in the multivariate logistic regression model** and added a **table in Methods** listing these variables (clinical, procedural, and sonographic), as requested.
- Adjusted ORs and 95% CI are now presented in **Results** in a regression summary table.

**3. Results, Tables, and Discussion**

**Reviewer comment:**

 Table 1 formatting can be improved (alignment of percentages and p-values). In the discussion section, clarify that “time-to-consultation” is an indicator of clinical presentation dynamics, not disease progression. A brief discussion on the implications for low-resource settings where hysteroscopy access is limited, can be added. Rather than claiming ET as a “robust prognostic marker,” the phrase “a potential surrogate marker reflecting disease burden” is suggested. Similarly, the phrase “cornerstone parameter” may be moderated to “key non-invasive parameter.” Formatting and minor errors also needs rectification, such as, correct “8- year and 11-month” to “8 years and 11 months” and “On 6 Mars 2025” to “on March 6, 2025.”

**Response:**

- **Table 1** has been reformatted for proper alignment of percentages and p-values.
- In the **Discussion**, we clarified that **time-to-consultation is an indicator of clinical presentation dynamics**, not disease progression.
- A brief discussion has been added on **implications for low-resource settings**, highlighting that ET measurement by TVUS may guide triage and reduce reliance on hysteroscopy where access is limited.
- Terminology revised: “robust prognostic marker” → **“potential surrogate marker reflecting disease burden”**; “cornerstone parameter” → **“key non-invasive parameter”**.
- Minor formatting and typographical errors have been corrected: “8- year and 11-month” → **“8 years and 11 months”**, “On 6 Mars 2025” → **“on March 6, 2025”**.

We sincerely thank you for their valuable comments, which have helped us improve the clarity, rigor, and clinical relevance of our manuscript.
